# The prognostic importance of jaundice in surgical resection with curative intent for gallbladder cancer

**DOI:** 10.1186/1471-2407-14-652

**Published:** 2014-09-03

**Authors:** Xin-wei Yang, Jian-mao Yuan, Jun-yi Chen, Jue Yang, Quan-gen Gao, Xing-zhou Yan, Bao-hua Zhang, Shen Feng, Meng-chao Wu

**Affiliations:** Eastern Hepatobiliary Surgery Hospital, Second Military Medical University, Changhai Road 225, Shanghai, 200438 China; Department of General Surgery, The First People’s Hospital of Wujiang affliated Wujiang Hospital of Nantong University, Suzhou, China; Department of General Surgery, Branch of the first People’s Hospital of Shanghai, North Sichuang Road 1878, Shanghai, 200081 China

**Keywords:** Gallbladder cancer, Jaundice, Curative resection, Preoperative biliary drainage, Prognosis

## Abstract

**Background:**

Preoperative jaundice is frequent in gallbladder cancer (GBC) and indicates advanced disease. Resection is rarely recommended to treat advanced GBC. An aggressive surgical approach for advanced GBC remains lacking because of the association of this disease with serious postoperative complications and poor prognosis. This study aims to re-assess the prognostic value of jaundice for the morbidity, mortality, and survival of GBC patients who underwent surgical resection with curative intent.

**Methods:**

GBC patients who underwent surgical resection with curative intent at a single institution between January 2003 and December 2012 were identified from a prospectively maintained database.

**Results:**

A total of 192 patients underwent surgical resection with curative intent, of whom 47 had preoperative jaundice and 145 had none. Compared with the non-jaundiced patients, the jaundiced patients had significantly longer operative time (p < 0.001) and more intra-operative bleeding (p = 0.001), frequent combined resections of adjacent organs (23.4% vs. 2.8%, p = 0.001), and postoperative complications (12.4% vs. 34%, p = 0.001). Multivariate analysis showed that preoperative jaundice was the only independent predictor of postoperative complications. The jaundiced patients had lower survival rates than the non-jaundiced patients (p < 0.001). However, lymph node metastasis and gallbladder neck tumors were the only significant risk factors of poor prognosis. Non-curative resection was the only independent predictor of poor prognosis among the jaundiced patients. The survival rates of the jaundiced patients with preoperative biliary drainage (PBD) were similar to those of the jaundiced patients without PBD (p = 0.968). No significant differences in the rate of postoperative intra-abdominal abscesses were found between the jaundiced patients with and without PBD (n = 4, 21.1% vs. n = 5, 17.9%, p = 0.787).

**Conclusions:**

Preoperative jaundice indicates poor prognosis and high postoperative morbidity but is not a surgical contraindication. Gallbladder neck tumors significantly increase the surgical difficulty and reduce the opportunities for radical resection. Gallbladder neck tumors can independently predict poor outcome. PBD correlates with neither a low rate of postoperative intra-abdominal abscesses nor a high survival rate.

## Background

The gallbladder is the most common site for biliary tract cancers. Most gallbladder cancer (GBC) patients have advanced disease at presentation, thus preventing curative resection and indicating poor prognosis [1–3]. However, recent advances in the understanding of its epidemiology and pathogenesis coupled with the development of newer diagnostic tools and therapeutic options have resulted in an enhanced optimism toward GBC management.

Curative resection provides the only chance for long-term survival [3]. However, most GBC patients have advanced disease at presentation because of late detection caused by non-specific symptomatology [4]. An aggressive tumor rapidly spreads in an anatomically "busy" area, making it unresectable [4]. Jaundice in GBC usually results from the infiltration of the extrahepatic bile duct by cancer and indicates advanced stage [1–3]. Numerous surgeons, especially those in Western countries, consider jaundice to be a contraindication of resection despite the consensus that surgical resection offers the only chance for long-term survival [4–6]. Furthermore, recent studies have shown that jaundice and extrahepatic bile duct involvement are independent predictors of poor outcome in GBC [3, 4, 7]. Resection is rarely recommended to treat advanced GBC [8, 9]. An aggressive surgical approach for advanced GBC remains lacking because of the association of this disease with serious postoperative complications and poor prognosis. Only a few studies have reported successful surgical resection of jaundiced GBC patients and evaluated the prognostic value of preoperative jaundice [2, 8, 9]. Most of these studies investigated small numbers of cases.

This study retrospectively analyzed the postoperative mortality, morbidity, and long-term survival of jaundiced and non-jaundiced GBC patients. This study aims to assess the safety and indications of curative resection in jaundiced GBC patients and to confirm that preoperative jaundice is not always a surgical contraindication.

## Methods

GBC patients who underwent surgical resection with curative intent at the Eastern Hepatobiliary Hospital institution between January 2003 and December 2012 were identified from a prospectively maintained hepatobiliary surgery database. Permission from the Second Military Medical University’s Institutional Review Board was obtained prior to data review. Written informed consents were obtained from all patients for surgical treatment and pathological examinations according to the institutional guidelines.

Surgical resection with curative intent was classified as either R0 or R1 [8]. According to the tumor–node–metastasis staging system of the International Union Against Cancer (UICC)/American Joint Committee on Cancer, 13 regional lymph nodes [gallbladder, pericholedochal, hepatic pedicle, proper hepatic artery (HA), and periportal nodes] were considered to be N1. Involvement of the periaortic, pericaval, superior mesenteric artery, and/or celiac artery lymph nodes were classified as N2. Involvement of inter-aortocaval lymph nodes was considered as M1 [4, 10]. Routine sampling of inter-aortocaval lymph nodes was not performed in the present study.

Between January 2003 and December 2012, 392 BGC patients were surgically treated at our unit, 192 of whom underwent resection with curative intent (overall curative resection rate: 48.9%). Of these 192 patients, 47 (24.5%) had preoperative jaundice. All patients who underwent either palliative or exploratory surgery were excluded from the analysis. Extensive invasion to the hepatoduodenal ligament, excessive presence of liver or peritoneal metastases beyond areas near the gallbladder, or bulky lymph node metastases were considered as surgical contraindications.

### Preoperative liver optimization

Patients were considered jaundiced when clinical jaundice was present upon initial examination and confirmed by elevated serum bilirubin level (>2.0 mg/dL). Of the 47 jaundiced GBC patients who underwent curative intent procedures, 19 underwent preoperative biliary drainage (PBD). Percutaneous transhepatic biliary drainage (PTBD) was performed in 10 patients (10/19; 52.6%). Endoscopic biliary drainage was performed in 9 patients (9/19; 47.4%). This intervention was performed approximately a week before hepatectomy based on the revaluation of hepatic function and the selection of the surgeon. No patient underwent preoperative portal vein embolization.

### Operative procedures

**Figure 1:**
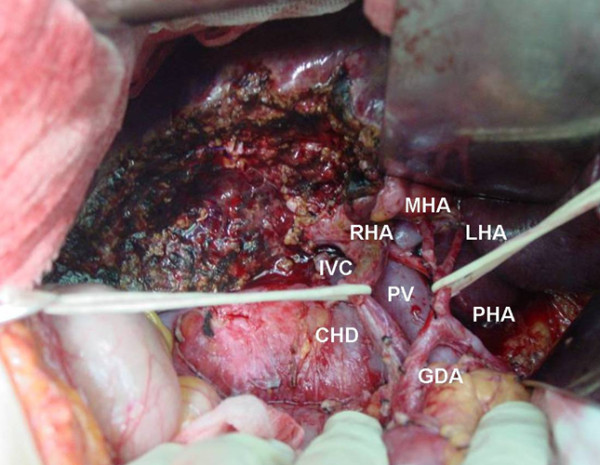
**Typical operative field after wedge resection with a 2 cm margin (including segments IVb/V) and skeletonization of the hepatoduodenal ligament.** We state that the subject of the photograph has given written informed consent by the patient to publication of the photograph. PV, portal vein; IVC, inferior vena cava; PHA, proper hepatic artery; RHA, right hepatic artery; LHA, left hepatic artery; MHA, middle hepatic artery; GDA, gastroduodenal artery

**Figure 2:**
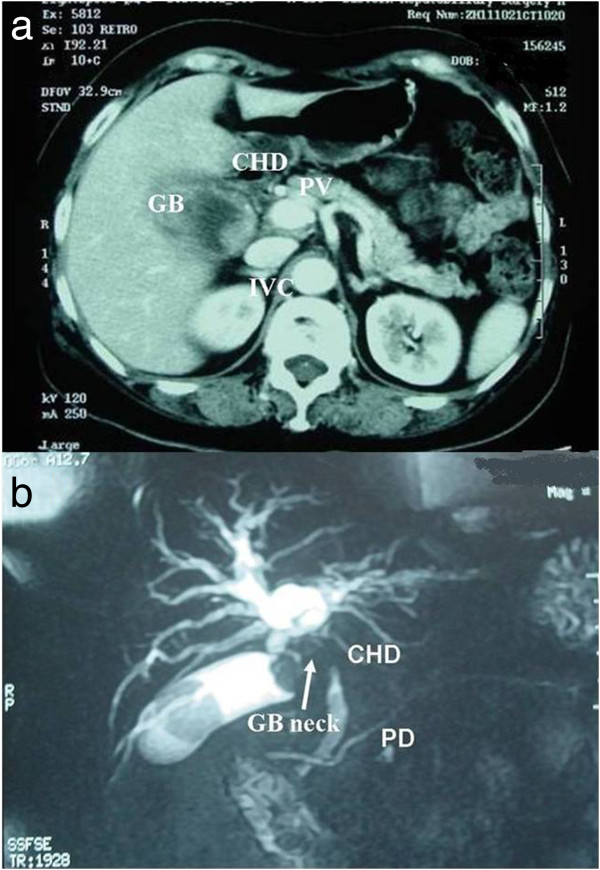
**Typical imaging feature of gallbladder carcinoma involving the hepatic hilum. a**. Enhanced CT shows gallbladder carcinoma with hepatic invasion. **b**. Preoperative MRCP shows that gallbladder carcinoma is located in the neck invading the hepatic hilum. The right hepatic artery and the common bile duct were involved by tumor in the surgery. We state that the subject of the photograph has given written informed consent by the patient to publication of the photograph. GB, gallbladder, CHD, common hepatic duct; IVC, inferior vena cava; PV, portal vein; PD, pancreatic duct.

Our center’s surgical policy for GBC involves radical surgery. For radical surgery, a partial hepatectomy with en bloc resection of GB and a dissection of regional lymph nodes (lymph nodes along the hepatoduodenal ligament and common HA and behind the pancreatic head) were routinely conducted (Figure 1). Partial hepatectomy includes extended right/left hepatectomy, right trisectionectomy, or wedge resection with a 2 cm margin (including segments IVb/V). Even advanced GBC was considered a candidate for resection as long as it could be anatomically removed. Extrahepatic bile duct resection was performed when the preoperative diagnostic images showed a tumor affecting the extrahepatic bile duct or when a tumor was significantly close to or invaded the extrahepatic bile duct upon intraoperative inspection (Figure 2).

Macroscopically involved adjacent organs were resected en bloc using major hepatectomy, pancreatoduodenectomy, partial gastrectomy, partial duodenal resection, partial colon resection, and/or portal vein/hepatic arterial resection and reconstruction as long as R0 resection was expected.

Surgeons assisted pathologists to correctly identify resection margins during the preparation of sections in fixed specimens. Surgical resection was considered to have curative intent (R0 or R1) when the whole tumor was resected, such that no macroscopically residual tumor could be detected. Stage grouping was performed according to the pTNM classification system of UICC, 7th edition [10].

Adjuvant therapy was given to 28 patients: intraoperative chemotherapy in 18 patients, postoperative chemotherapy in 15 patients (including a combination of intraoperative and postoperative chemotherapy in 13), postoperative radiotherapy in 15 patients, and a combination of chemotherapy and radiotherapy in 7 patients.

### Statistical analysis

The overall survival was measured from the date of operation to death, including deaths caused by cancer or other causes, or until the last day of follow-up. The two groups were compared using Student’s t test for parametric data and the Mann-Whitney U test for non-parametric data. The Chi-square test was used for categorical data. Survival curves were generated using the Kaplan–Meier method and compared using the log-rank test. Cox regression analysis was performed to determine which factor is the best prognostic determinant. Statistical significance was considered at p < 0.05. Statistical analyses were performed using SPSS Version 17.0 for Windows (SPSS, Inc., Chicago, IL, USA).

## Results

### Demographic data

Of the 192 GBC patients managed with curative intent during the 10-year inclusion period, 47 were jaundiced, of whom 21 were men and 26 were women. The median age of the jaundiced patients was 57.5 years (range: 35 - 80 years). No significant differences in risk factors and in-hospital mortality were observed between the jaundiced and non-jaundiced patients (Table 1). Microscopic invasion of the liver parenchyma and lymph node metastasis were more frequent in the jaundiced patients than in the non-jaundiced patients, but the difference was not significant (p = 0.183 and p = 0.091). An advanced T category was associated with preoperative jaundice (p = 0.019), suggesting more serious local tumor invasions in the jaundiced patients than in the non-jaundiced patients. More intra-operative bleeding and longer operative times were observed in the jaundiced patients than in the non-jaundiced patients (p = 0.001 and p < 0.001, respectively). This result suggests that more lesion resections were performed on the jaundiced patients than on the non-jaundiced patients. Hence, the combined resection of adjacent organs (CRAO) was more frequent in the jaundiced patients than in the non-jaundiced patients to achieve curative resection (p = 0.001). However, the R0 resection rates were similar between the jaundiced and non-jaundiced patients (p = 0.068).

**Table 1:** **Demographic data of jaundiced (n = 47) and non-jaundiced GBC patients (n = 145)**

	Jaundiced, n	Non-jaundiced, n	p-value
Male gender	21	45	0.088
Mean age (range)	57.5 ± 11.0(35-80)	58.2 ± 10.2(23-83)	0.725
Postoperative hospital stay	19.7 ± 13.3 (4–85)	11.5 ± 6.4 (5–51)	< 0.001
Associated gallbladder disease			0.983
Gallstones	26 (55.3%)	80 (55.2%)	
Gallbladder polyp	1 (2.1%)	3 (2.0%)	
Nil	20 (42.6%)	62 (42.8%)	
Tumor location			< 0.001
Gallbladder neck	28 (59.6)	26 (17.9)	
Gallbladder body	10 (21.3)	70 (48.3)	
Gallbladder fundus	9 (19.1)	49 (33.8)	
Histologic type			0.259
Well differentiated	2 (4.3)	9 (6.2)	
Moderately differentiated	41 (87.2)	109 (75.2)	
Poorly differentiated	4 (8.5)	27 (18.6)	
Extent of liver resection			0.590
Major hepatectomy (>3 segments)	4	4	
Anatomical segments IV-V	8	42	
Gallbladder bed	35	99	
Extrahepatic bile duct resection	44 (93.6)	12 (8.3%)	< 0.001
Pathologic extrahepatic bile duct invasion	38 (80.9)	12 (8.3%)	< 0.001
Combined resection of adjacent organs	11 (23.4%)	4 (2.8%)	< 0.001
Hepatic invasion	26 (55.3%)	64 (44.1%)	0.183
Lymph node metastasis	30 (63.8%)	72 (49.7%)	0.091
Vascular invasion	2 (4.3)	3 (2.1%)	0.415
pT			0.019
pT1-2	16 (34.1%)	78 (53.8%)	
pT3-4	31 (65.9%)	67 (46.2%)	
Intra-operative bleeding (mL)	754.3 ± 600.9 (200–3200)	386.0 ± 251.8(200–1800)	0.001
Operative time (min)	310.0 ± 82.7 (100–470)	224.3 ± 77.3(100–400)	< 0.001
R0	36 (76.6%)	127 (87.6%)	0.068
Mortality (number of patients)	3 (6.4%)	3 (2.1%)	0.141
Morbidity (need invasive treatment)	16 (34.0%)	18 (12.4%)	0.001

Note that adjacent organs include the pancreas, duodenum, stomach, and/or colon other than the liver and extrahepatic bile duct.

### Surgical procedures

Table 1 summarizes the surgical procedures. In this study, "major hepatectomy" indicates right or left hepatectomy, extended right or left hepatectomy, or right or left trisegmentectomy while "minor hepatectomy" indicates segmental resection or less. Parenchymal transection was performed under HA and portal vein clamping for 15 min at 5 min intervals. Hepatectomy was performed in all 192 patients. Eight patients (8.5%) underwent major hepatectomy, including one patient with combined caudate lobe resection, three patients with partial portal vein (PV) resection, and two patients with HA resection and reconstruction. The following combined resections of other organs were performed in 15 patients: pancreatoduodenectomy (n = 2), wedge duodenal resection (n = 1), segmental colon resection (n = 1), partial kidney resection (n = 1), and partial gastrectomy (n = 10).

All patients underwent en bloc dissection of the regional lymph nodes (lymph nodes along the hepatoduodenal ligament and common hepatic artery and behind the pancreatic head). Morbidity was significantly lower in the non-jaundiced patients than in the jaundiced patients (12.4% vs. 34.0%, p = 0.001). Compared with the non-jaundiced patients, the jaundiced patients had significantly longer operative times (p < 0.001) and more intra-operative bleeding (p = 0.001), advanced T category (p = 0.019), common bile duct resections (93.6% vs. 8.3%, p < 0.001), extensive pathologic extrahepatic bile duct invasion (pEBI) (80.9% vs. 8.3%, p = 0.001), and frequent CRAOs (23.4% vs. 2.8%, p = 0.001) (Table 1). However, no significant differences in major hepatectomies and R0 resection were observed between the jaundiced and non-jaundiced patients (8.5% vs. 2.8%, p = 0.590 and 76.6% vs. 87.6%, p = 0.068).

### Mortality and morbidity in 192 GBC patients who underwent curative intent procedures (Table 2)

**Table 2:** **Univariate and multivariate analyses for hospital mortality in GBC patients who underwent surgical resection with curative intent (n = 192)**

Variables		No. of patients	Morbidity	Univariate	Multivariate	
				p-value	RR (95% CI)	p-value
Gender				0.128		
	Female	126	18 (14.3%)			
	Male	66	16 (24.2%)			
Age				0.505		
	< 60 years	107	17 (15.9%)			
	≥ 60 years	85	17 (20.0%)			
Preoperative jaundice				0.034	0.387 (0.164–0.916)	0.031
	Present	47	16 (34.0%)			
	Absent	145	18 (12.4%)			
Major hepatectomy				0.183		
	Yes	8	4 (50.0%)			
	No	184	30 (16.3%)			
Extrahepatic bile duct resection				0.717		
	With	56	17 (30.4%)			
	Without	136	17 (12.5%)			
Combined resection of adjacent organs (CRAO)				0.168		
	With	15	7 (46.7%)			
	Without	177	27 (15.3%)			
Combined portal vein/hepatic artery resection				0.802		
	With	5	1 (20.0%)			
	Without	187	33 (17.6%)			
Operative time 0.280						
	< 240 min	87	9 (10.3%)			
	≥ 240 min	105	25 (23.8%)			
Intraoperative blood infusion 0.056					0.420 (0.175-1.007)	0.052
	With	44				
	Without	148				

Adjacent organs include the pancreas, duodenum, stomach, and/or colon other than the liver and bile duct.

The postoperative mortality and morbidity rates of the 192 GBC patients were 3.1% (n = 6) and 17.7% (n = 34), respectively. Postoperative complications were graded I in 2 patients, II in 12, IIIa in 11, IIIb in 2, IVa in 1, and V in 6 patients using Clavien–Dindo classification [11]. Considering the lack of a significant difference in mortality between the jaundiced patients and non-jaundiced patients (p = 0.141), we focused on morbidity. Univariate analysis showed that the risk factors for morbidity were preoperative jaundice (34.0% vs. 12.4%, p = 0.034) and intraoperative blood transfusion (34.1% vs. 12.8%, p = 0.056). Multivariate analysis revealed that preoperative jaundice was the only independent predictor of postoperative morbidity.

### Mortality and morbidity in 47 jaundiced GBC patients (Table 3)

**Table 3:** **Univariate analyses for hospital mortality in GBC patients with preoperative jaundice (n = 47)**

Variable		No. of patients	Morbidity	Univariate
				p-value
Gender				0.927
	Female	26	14 (36.8%)	
	Male	21	2 (22.2%)	
Age				0.927
	< 57 years	26	6 (31.6%)	
	≥ 57 years	21	10 (35.7%)	
pEBI				0.405
	Yes	38	2 (50.0%)	
	No	9	14 (32.6%)	
Preoperative biliary drainage				0.769
	Yes	19	15 (34.1%)	
	No	28	1 (33.3%)	
Major hepatectomy				0.481
	Yes	4	5 (45.5%)	
	No	43	11 (30.6%)	
Extrahepatic bile duct resection				0.979
	With	44	1 (50.0%)	
	Without	3	15 (33.3%)	
CRAO				0.361
	With	11	8 (44.4%)	
	Without	36	8 (27.6%)	
Combined portal vein/hepatic artery resection				0.626
	With	2		
	Without	45		
Operative time				0.236
	< 300 min	18	8 (32.0%)	
	≥ 300 min	29	8 (36.4%)	
Intraoperative blood infusion				0.753
	With	25	8 (32.0%)	
	Without	22	8 (36.4%)	

Note that adjacent organs include the pancreas, duodenum, stomach, and/or colon other than the liver and bile duct.

The postoperative mortality and morbidity rates in the jaundiced patients were 6.4% (n = 3) and 34.0% (n = 16), respectively. The causes of death were acute liver failure (n = 1) followed by renal failure; intra-abdominal bleeding (n = 1); and sepsis with multiorgan failure (n = 1).

Postsurgical complications were detected in 16 jaundiced patients. The most frequent complications were intra-abdominal abscesses (n = 9), biliary leakage (n = 2), intra-abdominal bleeding (n = 3), aspiration pneumonia (n = 1), and liver failure (n = 1). These complications required an invasive procedure in 13 patients (reoperation: n = 2, ultrasound guided drainage: n = 11). The average postoperative hospital stay of the jaundiced patients was 19.7 d (range: 4-85 d), which was longer than that of the non-jaundiced patients (p < 0.001). Univariate analysis identified no risk factor for postoperative morbidity among the jaundiced patients.

### Survival and risk factors in 192 GBC patients who underwent curative intent procedures (Tables 4 and 5)

**Table 4:** **Univariate analysis of 14 variables related to survival of GBC patients who underwent surgical resection with curative intent (n = 192)**

Variable	Cutoff level	Number	Survival rates (%)		p-value
			3 year	5 year	
Age (year)					0.012
	< 60	107	43.2	39.0	
	≥ 60	85	25.5	15.3	
Sex					0.805
	Male	66	34.9	24.4	
	Female	126	35.9	29.7	
Jaundice					< 0.001
	Present	47	6.0	6.0	
	Absent	145	42.9	36.0	
Associated gallstone					0.577
	Present	106	35.3	25.8	
	Absent	86	35.3	30.9	
Curability					< 0.001
	Curative	163	40.4	32.1	
	Non-curative	29	7.9	7.9	
Tumor location					< 0.001
Gallbladder neck		54	12.7	9.5	
Gallbladder body/fundus		138	44.0	35.9	
pT (TNM)					< 0.001
	pT1 and 2	95	57.4	49.5	
	pT3 and 4	97	12.8	6.4	
Lymph node metastasis					< 0.001
	Negative	90	55.1	43.5	
	Positive	102	17.5	14.6	
Histologic differentiation					0.154
Well/Moderate		161	36.0	30.8	
	Poor	31	32.1	11.5	
Hepatic invasion					< 0.001
	Present	90	14.2	6.7	
	Absent	102	53.1	45.9	
CRAO					< 0.001
	Present	15	0.0	0.0	
	Absent	177	36.0	28.7	
Combined portal vein/hepatic artery resection					0.041
	Present	5	0.0	0.0	
	Absent	187	37.8	30.1	
Intraoperative blood infusion					0.011
	Present	44	16.6	5.5	
	Absent	148	41.1	35.2	
Adjuvant therapy					0.782
	Yes	28	31.6	13.2	
	No	164	35.8	30.4	
	Overall	192	35.3	28.1	

**Table 5:** **Results of multivariate analysis**

Variable	Regression coefficient	Standard error	p-value	Relative risk	95% CI
Age	0.287	0.189	0.129	1.332	0.920–1.928
Jaundice	-0.289	0.277	0.295	0.749	0.435–1.287
Curability	0.166	0.260	0.523	1.181	0.709–1.965
Tumor location	-0.691	0.231	0.003	0.501	0.318–0.788
pT	0.292	0.416	0.482	1.339	0.593-3.026
Lymph node metastasis	-0.453	0.225	0.044	0.636	0.409–0.987
Hepatic invasion	-0.641	0.384	0.095	0.527	0.248–1.119
CRAO	-0.013	0.359	0.972	0.987	0.489–1.994
Combined portal vein/hepatic artery resection	-0.741	0.563	0.188	0.477	0.158–1.437
Intraoperative blood infusion	-0.347	0.246	0.159	0.707	0.436–1.145

**Figure 3:**
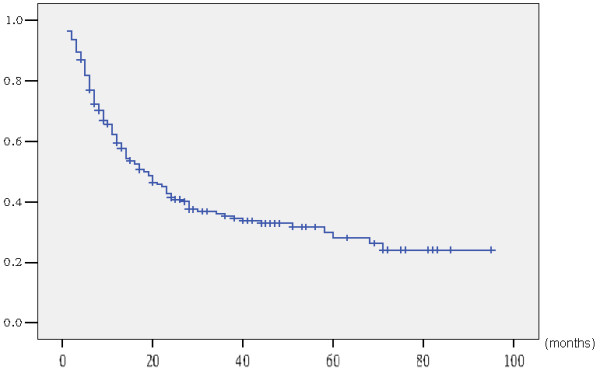
**Actuarial survival curve of 192 gallbladder cancer patients following surgical resection with curative intent.**

**Figure 4:**
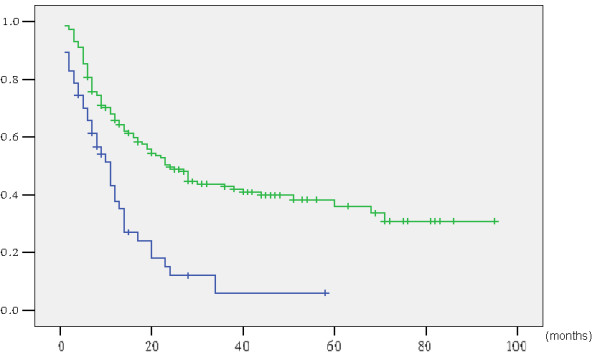
**Actuarial survival curve according to preoperative jaundice (with vs. without jaundice: p < 0.001).**

Overall, the three- and five-year survival rates and median survival time for the 192 patients were 35.3%, 28.1%, and 37.0 months, respectively (Figure 3). Survival curves for the 192 patients, grouped according to preoperative jaundice status, are shown in Figure 4. The five-year survival rate and median survival time were 6.0% and 14.0 months for the 47 jaundiced patients, respectively, and 36.0% and 43.0 months for the 145 non-jaundiced patients, respectively. The jaundiced patients had significantly lower survival rates than the non-jaundiced patients (p < 0.001).

Univariate and multivariate analyses were performed on the 192 GBC patients who received surgical resection with curative intent to identify the factors that influence long-term survival (Tables 4 and 5). Univariate analysis revealed that the significant risk factors of survival were age (p = 0.012), preoperative jaundice (p < 0.001), curative resection (p < 0.001), tumor location (p < 0.001), pT factor (p < 0.001), lymph node metastasis (p < 0.001), hepatic invasion (p < 0.001), CRAO (p < 0.001), combined portal vein/hepatic artery resection (p = 0.041), and intraoperative blood transfusion (p = 0.011) (Tables 4). Multivariate analysis was performed to determine which univariate prognostic relationships are independent predictive factors. Lymph node metastasis and tumors at the gallbladder neck were the significant risk factors of poor prognosis in this analysis (Table 5).The patients who underwent CRAO had significantly lower survival rates than those who did not undergo the procedure (p < 0.001). The patients who underwent R0 resection had a higher five-year survival rate than those who underwent R1 resection (p < 0.001). However, multivariate analysis revealed that neither CRAO nor R1 resection was an independent predictor of poor prognosis. The jaundiced patients had significantly lower survival rates than the non-jaundiced patients (p < 0.001) (Figure 4). However, multivariate analysis demonstrated that preoperative jaundice was not a significant risk factor (p = 0.295).

### Survival and risk factors in 47 jaundiced GBC patients (Table 6)

**Table 6:** **Univariate and multivariate analyses of 14 variables related to survival of GBC patients with preoperative jaundice (n = 47)**

Variable	Cutoff level	Number	Survival rates (%)		Univariate	Multivariate	
			1 year	3 year	p-value	RR (95% CI)	P
Age (year)					0.980		
	< 60	26	42.1	8.0			
	≥ 60	221	25.5	15.3			
Sex					0.477		
	Male	21	36.6	24.4			
	Female	26	39.6	29.7			
Preoperative biliary drainage					0.968		
	Present	19	37.9	6.3			
	Absent	28	38.5	8.7			
Associated gallstone					0.341		
	Present	26	53.8	11.5			
	Absent	21	54.5	0.0			
Curability					0.004	2.845 (1.314–6.158)	0.008
	Curative	36	47.0	7.2			
	Non-curative	11	9.1	0.0			
Tumor location					0.699		
Gallbladder neck		28	42.5	8.5			
Gallbladder body/fundus		19	30.7	0.0			
pT (TNM)					0.170		
	pT1 and 2	16	42.2	12.7			
	pT3 and 4	31	35.2	4.4			
Lymph node metastasis					0.328		
	Negative	17	47.1	7.8			
	Positive	30	32.2	10.1			
Histologic differentiation					0.432		
Well/Moderate		43	38.8	6.0			
	Poor	4	25.0	0.0			
Hepatic invasion					0.728		
	Present	26	30.5	6.1			
	Absent	21	46.2	8.8			
CRAO					0.728		
	Present	11	43.6	0.0			
	Absent	36	35.9	7.2			
Intraoperative blood infusion					0.893		
	Present	25	34.5	12.9			
	Absent	22	41.5	0.0			
Adjuvant therapy					0.179		
	Yes	7	51.4	25.7			
	No	40	35.6	4.9			
	Overall	47	37.8	6.0			

The one- and three-year survival rates and median survival time of the 47 jaundiced patients were 37.8%, 6.0%, and 14.0 months, respectively. Univariate analysis was performed on the 47 jaundiced patients who received surgical resection with curative intent to identify the factors that influence the long-term survival of these patients (Table 6). The patients who underwent R0 resection survived longer than those who underwent R1 resection (p = 0.004). Multivariate analysis showed that R1 resection was the only independent predictor of poor prognosis.

### Comparison between jaundiced patients with and without PBD (Table 7)

**Table 7:** **Compared analyses for jaundiced patients with and without PBD (n = 47)**

Variables		With PBD,n = 19	Without PBD, n = 28	p-value
Male gender		6	15	0.141
Mean age (range)		55.2 ± 11.1	59.0 ± 10.9	0.247
Postoperative hospital stay		18.8 ± 9.1	20.4 ± 15.6	0.689
Bilirubin level at presentation		234.0 ± 120.5	194.4 ± 125.7	0.287
Intra-operative bleeding (mL)		800.0 ± 761.4	723.2 ± 475.6	0.672
Operative time (min)		328.9 ± 77.7	297.1 ± 84.8	0.199
R0		15 (78.9)	21 (75.0)	0.795
Mortality (number of patients)		1 (5.3%)	2 (7.1%)	0.854
Morbidity (need invasive treatment)		6 (31.6%)	10 (35.7%)	0.755
Intra-abdominal abscesses		4 (21.1%)	5 (17.9%)	0.787
Survival				0.968
	One-year survival	37.9%	38.5%	
	Three-year survival	6.3%	8.7%	

After PBD, direct bilirubin decreased from 216.5 ± 131.9 mol/L to 116.9 ± 66.3 mol/L (p < 0.001) with decreasing AST and ALT levels. Overall, the one- and three-year survival rates were 37.9% and 6.3% in the jaundiced patients with PBD (n = 19), respectively, and 38.5% and 8.7% in the jaundiced patients without PBD (n = 28), respectively. The primary endpoint of the three-year survival after surgery was not significantly different between the groups. The survival rates of the jaundiced patients with PBD were similar to those of the jaundiced patients without PBD (p = 0.968). The only patient who died of liver failure within the first 30 d postsurgery was a jaundiced GBC patient without PBD. The rate of postoperative intra-abdominal abscesses was not significantly different between the jaundiced patients with and without PBD (n = 4, 21.1% vs. n = 5, 17.9%, p = 0.787).

## Discussion and conclusions

Preoperative jaundice is an indicator of advanced GBC with poor prognosis [1–4]. The present study confirmed that jaundiced patients had more postoperative complications (34.0% and 12.4%, p = 0.001) and lower five-year survival rates than non-jaundiced patients (6.0% and 36.0%, p < 0.001). However, multivariate analysis showed that preoperative jaundice was not a significant risk factor of poor outcome (p = 0.295). The present study involved 192 GBC patients who underwent resection with curative intent. R0 resection was performed in 163 patients. This study is one of the largest investigations that have ever been published.

### Implication of PBD before resection for advanced GBC with preoperative jaundice

Preoperative liver optimization has been a subject of debates for the last two decades, especially in PBD [12]. A prospective cohort study found that PBD significantly increases the rate of infectious complications [12]. Another study concluded that the routine use of PBD was not justified, considering that the mortality rate was not significantly different and the hepatic synthetic function recovery was identical to those of non-jaundiced patients [13]. To date, a few randomized controlled trials or meta-analyses have been conducted to systematically evaluate the value of PBD for the surgical resection of advanced GBC with preoperative jaundice. In the present study, the only patient who died of liver failure was a jaundiced GBC patient without PBD. Moreover, the postoperative mortality rates were not significantly different between the jaundiced patients with and without PBD.

Biliary obstruction is associated with renal failure, body fluid disturbances, and myocardial dysfunction. In our population, preoperative jaundice was a significant prognostic factor (p < 0.001) (Figure 4), which was consistent with the results our previous study [14]. We proposed that PBD via PTBD improves preoperative liver function; however, the effect of PBD on postoperative infection risk remains to be clarified [15–19]. In the present study, the decrease in the bilirubin level was statistically relevant (p < 0.001). However, this benefit was not associated with a longer survival time (p = 0.968).

Intra-abdominal abscesses often directly precede liver failure; thus, several researchers have shown that PTBD and increased intra-abdominal abscesses are significantly related [12, 16]. However, no significant differences in the rates of postoperative intra-abdominal abscesses were detected between jaundiced patients with and without PBD (21.1% vs. 17.9%, respectively, p = 0.787). Univariate analysis showed that PBD was not a risk factor for postoperative complications in jaundiced patients.

Liver surgery in jaundiced patients is supposed to have particular risks because of the hepatic and systemic changes caused by hyperbilirubinemia [15, 18]. Experimental studies on jaundiced animals have shown the benefits of biliary drainage, especially of internal biliary drainage with the restoration of biliary salt enterohepatic circulation [20]. PBD should increase cholestatic liver tolerance to ischemia [21] and reduce blood loss [22]. However, such benefits were not observed through the postoperative mortality and morbidity rates in jaundiced patients, which was not consistent with the results of previous studies [12, 23]. The lack of postoperative liver failure in the present study may be attributed to the low frequency of additional major surgical procedures, such as major hepatectomy and pancreaticoduodenectomy.

### Preoperative jaundice as an indicator of poor prognosis and high postoperative morbidity but not a surgical contraindication

Jaundice is an indicator of advanced GBC with poor prognosis [1–4]. In the present study, the jaundiced patients had longer postoperative hospital stay and lower five-year survival rates than the non-jaundiced patients (6.0% and 36.0%, respectively). However, multivariate analysis revealed that preoperative jaundice was not a significant risk factor of poor outcome. Therefore, we have not solely considered jaundice to be a surgical contraindication. Several jaundiced patients had increased survival rates following resection. However, in the present study, preoperative jaundice was the only independent predictor of postoperative morbidity in GBC patients. Clinicians should be aware of this finding.

Several recent articles have been published with encouraging results. Agarwal et al. [14] reported that the presence of obstructive jaundice caused by GBC is neither a sole indicator of advanced disease nor a surgical contraindication. D’Angelica et al. [4] reported a five-year survival rate of 20% and a median survival time of 19 months after resection of GBC with clinical common bile duct involvement. They concluded that advanced GBC patients with preoperative jaundice and/or pEBI are candidates for resection when distant metastases are absent and R0 resection is achievable. This finding is consistent with the results of Hideki Nishio [9] and our previous report [24]. In the present study, the non-jaundiced patients, even those with locally advanced (T3/T4) GBC, exhibited good prognosis. Our data are consistent with the findings of Nishio et al. [9] and Agarwal et al. [14]. Multivariate analysis showed that non-curative resection was the only independent predictor of poor prognosis in jaundiced GBC patients. Therefore, even advanced GBC patients with preoperative jaundice should be resected whenever R0 resection is achievable.

**Figure 5:**
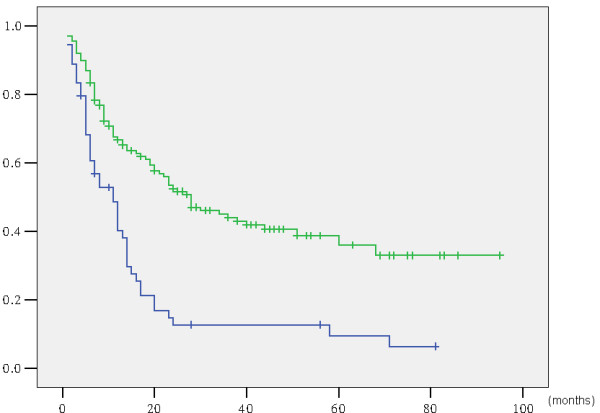
**Actuarial survival curve according to tumor location (gallbladder neck vs. gallbladder body/fundus: p < 0.001).**

In the present study, multivariate analysis of all 192 GBC patients revealed that lymph node metastasis and gallbladder neck tumors were independent prognostic factors. Therefore, an extended dissection of the regional lymph nodes must be routinely performed to achieve good prognosis. However, no specific patterns between recurrence incidence and gallbladder neck tumors were found. In addition, the mechanism by which gallbladder neck tumors contribute to GBC progression remains unknown. One possible explanation is that the gallbladder neck is in an anatomically "busy" area because of involved adjoining bile duct, portal vein, liver, duodenum, and colon, making surgical resection and radiotherapy difficult [24, 25]. Gallbladder neck tumors significantly increase surgical difficulty and reduce the opportunities for radical resection. Meanwhile, a relatively small tumor in the gallbladder neck infiltrates the hepatic hilum, thus causing obstructive jaundice [26]. In conclusion, advanced GBC with extrahepatic bile duct invasion and/or jaundice is a candidate for resection when R0 resection is achievable. However, the radical resection of advanced gallbladder neck cancer is still challenging with high postoperative morbidity and poor prognosis (Figure 5).

### Extended resection for advanced GBC

Treatment of advanced GBC requires combined resection because it usually extends into adjacent organs, such as the liver, transverse colon, duodenum, extrahepatic bile duct, HA, and PV. For advanced GBC, extended resections that include extended hepatectomy, pancreatoduodenectomy, extended lymph node dissection, and combined vascular resection have been recently performed [27–29]. Extended resections have a high surgical mortality rate, and patients who have undergone such surgery remain to have poor prognosis [30–32]. The benefit of radical resection is apparently limited in advanced GBC patients. Hence, identifying patients who will potentially benefit from aggressive resection is important. Understanding the limits of the current surgical therapies may save patients with aggressive tumors from high morbidity and mortality rates related to surgeries that are unlikely to offer any benefit. It also allows patients and physicians to focus on palliation to improve the patients’ quality of life.

CRAO other than the liver and extrahepatic bile duct was an independent predictor of poor prognosis (p = 0.041). No three-year survivors were found among the patients undergoing CRAO, although CRAO was not a significant risk factor for poor outcome in multivariate analysis (Table 5). Therefore, aggressive resection should be carefully planned in patients who require CRAO. Our data are also consistent with the findings of Nishio [9].

Considering the high risk of death and poor long-term survival, we found no definitive benefit from the extended resections for advanced GBC [30]. A parenchymal preservation strategy has been investigated on the basis of the results published by Agarwal et al. [14]. A similar strategy for minor resection has been recently recommended by Regimbeau [8] for jaundiced patients with GBC. In the present study, we retained the most normal liver parenchyma to reduce the incidence of postoperative complications from R0 resection. We also obtained a comparable five-year survival rate and relatively low mortality and morbidity rates. Therefore, we recommend minor resection when a negative margin is ensured during operation.

This study has some limitations. Despite the large number of patients included in the present study, the patients were a highly selected cohort and represent only a fraction of the total number of jaundiced GBC patients diagnosed during the study period. Additional investigations with a large patient population in a multicenter study are needed before definitive conclusions can be drawn. Further studies must be conducted to clarify the resection contraindications in advanced GBC patients.

### Ethical standards

All studies were approved by the Committee on Ethics of Second Military Medical University.

## Authors’ information

Yuan Jian-mao and Chen Jun-yi are the co-first author.
